# Photoconductivity, pH Sensitivity, Noise, and Channel Length Effects in Si Nanowire FET Sensors

**DOI:** 10.1186/s11671-018-2494-5

**Published:** 2018-03-27

**Authors:** Ferdinand Gasparyan, Ihor Zadorozhnyi, Hrant Khondkaryan, Armen Arakelyan, Svetlana Vitusevich

**Affiliations:** 1 Bioelectronics (ICS-8), Forschungszentrum Jülich, 52425 Jülich, Germany; 2 Yerevan State University, 1 Alex Manoogian St., 0025 Yerevan, Armenia

**Keywords:** Nanowire field-effect transistors, Biochemical, Photoexcitation, Channel length effects

## Abstract

Silicon nanowire (NW) field-effect transistor (FET) sensors of various lengths were fabricated. Transport properties of Si NW FET sensors were investigated involving noise spectroscopy and current–voltage (I–V) characterization. The static I–V dependencies demonstrate the high quality of fabricated silicon FETs without leakage current. Transport and noise properties of NW FET structures were investigated under different light illumination conditions, as well as in sensor configuration in an aqueous solution with different pH values. Furthermore, we studied channel length effects on the photoconductivity, noise, and pH sensitivity. The magnitude of the channel current is approximately inversely proportional to the length of the current channel, and the pH sensitivity increases with the increase of channel length approaching the Nernst limit value of 59.5 mV/pH. We demonstrate that dominant 1/f-noise can be screened by the generation-recombination plateau at certain pH of the solution or external optical excitation. The characteristic frequency of the generation-recombination noise component decreases with increasing of illumination power. Moreover, it is shown that the measured value of the slope of 1/f-noise spectral density dependence on the current channel length is 2.7 which is close to the theoretically predicted value of 3.

## Background

Over the past decade, nanosized silicon structures have been under intensive study [1] due to their promising electrical, optical, chemical, thermal, and mechanical properties. Compared to larger structures, nanoscale field-effect transistors (FETs) allow measuring electrical, optical, and other types of very small signals due to increased surface-to-volume ratio of the sample. The small sizes of nanostructures make them ideal for sensing of small sample volumes with low analyte concentrations. The features and properties of pH sensors are discussed more detailed in [2–4]. It is shown that the pH sensitivity of silicon bulk materials is poor. Good pH sensing properties of Si nanowires (NW), with a sensitivity of 58.3 mV/pH, were observed. For example, in the field of medical diagnostics, nanoscaled structures aiming the utilization of low-dimensional nanostructure such as carbon nanotubes, metallic, or semiconducting NW or atom-sized thin nanoribbons (NR) can be implemented for a variety of applications [5]. Among the mentioned structures, silicon NR and NW FET structures open prospects for label-free, real-time, and high-sensitive detection of biomolecules using affinity-based binding principles [6]. The sensitivity of different NR dimensions was studied. It was shown that the new sensor with integrated reference NR can be utilized for real-time error monitoring during pH sensing [6]. New features and functions are continuously added to the electronic devices, such as health monitoring mobile systems and wearable devices. Despite the success of such personal health monitoring systems [7], the next generation of wearable devices is expected to include also a portable “lab-on-a chip”—set of medical biosensors which can be used for the detection and diagnosis of various medical substances [8, 9]. In order to be able to monitor and detect the early stages of disease in ideal case at the level of single molecule, the size of the sensor transducer has to be comparable with the biological markers under test. Therefore, biosensors based on NWs and NRs have to be developed for the monitoring of biological events that occur at very small dimensions. Another important area of application is optoelectronic, where the light interaction with nanostructures may be used for future optical device applications. Sub-wavelength diameters and proximity effects may lead to advanced optical properties such as low reflectance and thus high absorption. Investigation results of Si NW optical absorption have demonstrated the strong size-dependent effects [10–12]. Studies of the broadband optical absorption showed increased total optical absorption spectra for Si NW samples [13]. Si NWs lead to a significant reduction of the reflectance compared to the solid silicon films [13, 14]. Optical absorption increases while the wavelength decreases. It should be noted that, unlike the bulk material, nanosized Si structures may be direct band gap semiconductors, which make them excellent choice for optical applications [11, 13, 15–18]. On the other hand, the size scaling increases the band gap [15]. This may result in a successful shift of the absorption spectra to short wavelengths [11, 18]. With size decrease, the limitations regarding current and voltage have to be also considered. For devices operating at weak signal levels, internal noise plays crucial role [4, 19–21]. It determines one of the most important parameters of sensors—signal-to-noise ratio (SNR). As it is shown for double-gated SiNW sensors, pH sensitivity increases with the liquid gate voltage and SNR has higher value (~ 10^5^) [11, 18]. The nanoribbon approach opens up for large scale CMOS fabrication of highly sensitive biomolecule chips for potential use in medicine and biotechnology [22].

State-of-the-art research on nanoscale materials has revealed that electronic, magnetic, thermal, and optical properties may differ dramatically when their one-dimensional forms are synthesized. Nanowires obtained by utilizing single- or few-atom-thick lamellar crystals are novel forms of one-dimensional nanoscale materials and are ideal systems for investigation of the size dependence of the fundamental properties.

A detailed analysis of the latest achievements on the methods of synthesis and theoretical studies of NR are presented in [23]. In the literature, photoconductivity, pH sensitivity, noise, and channel length effects in the same set of NW FET arrays have not been studied in detail. However, surface roughness and contribution of dielectric layers may considerably change structure properties depending on fabrication technology applied for different set of devices. In this respect, understanding channel length effects in the same set of NW FETs are important for the development of devices with advanced functionality.

The present work is devoted to the study of silicon nanowire-based FETs, including the sample fabrication technology, and chip characterization, their dark and light current–voltage (I–V) characteristics and pH sensitivity. Effects of channel length influence on the source-drain currents, pH sensitivity, and low-frequency noise are described. We demonstrate that silicon nanowires, fabricated on the basis of a thin silicon layer on an oxidized silicon substrate, can have high pH sensitivity fairly close to the Nernst limit.

## Methods/experimental

**Fig. 1:**
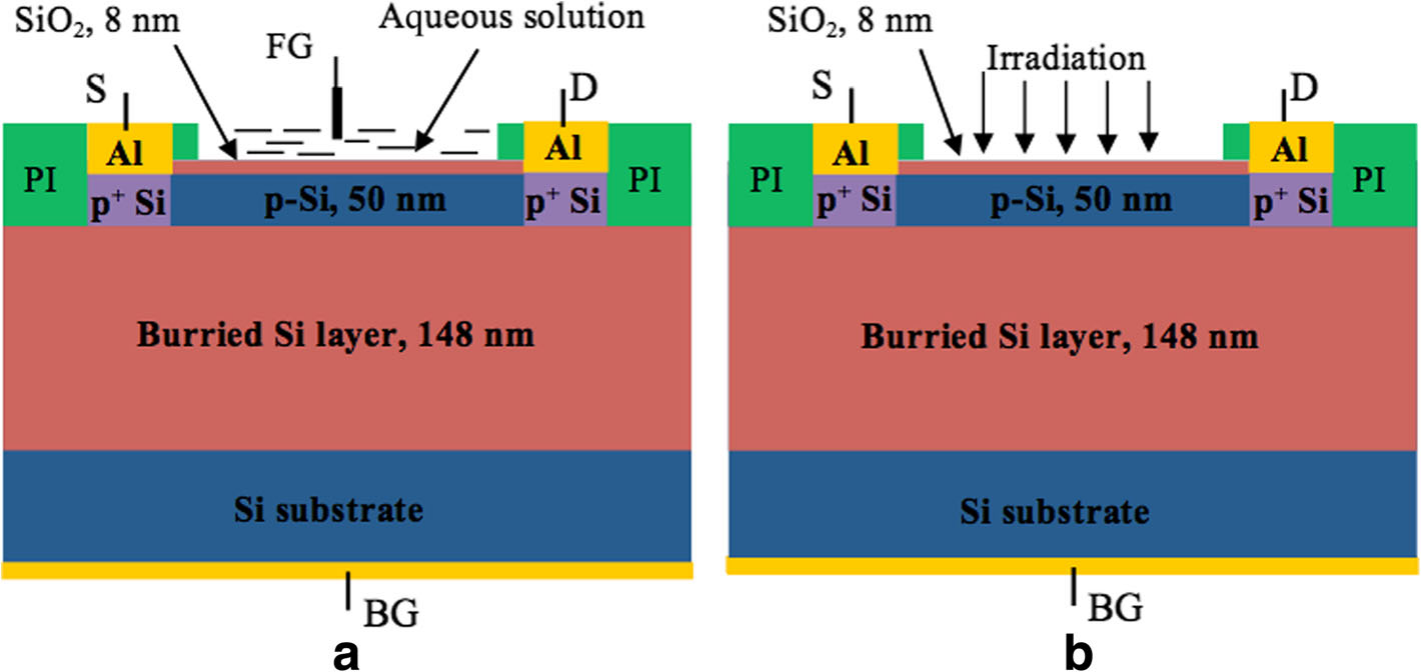
Si nanowire field-effect transistor structures under study. Schematic picture of the samples under study: pH—sensor operating mode (**a**) and photo-receiver operating mode (**b**). PI polyimide layer, S source, D drain, FG front gate (reference electrode, RE), BG back gate

**Fig. 2:**
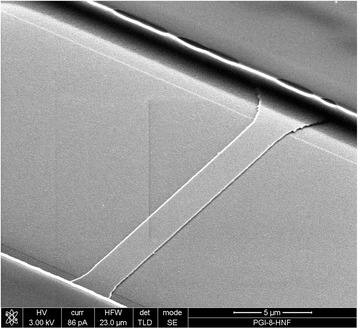
SEM image of Si NW FET structure. Typical scanning electron micrograph (SEM) image of fabricated Si nanoribbon field-effect transistor structure

Silicon NW structures were fabricated on the basis of silicon-on-insulator (SOI) wafers purchased from SOITEC. The process starts from the thermal oxidation to form 20-nm-thick silicon oxide hard masks. The active silicon layer thickness is 50 nm. NWs of various geometries are then patterned in hard mask using optical lithography and transferred in SiO_2_ layer using reactive ion etching process step. The pattern is utilized to obtain silicon nanoribbons and nanowires using wet chemical etching in the tetramethylammonium hydroxide (TMAH) solution. Gate dielectric layer, which also serves as a channel protection from liquid environment, is thermally grown 8-nm-thick silicon oxide. The NW channel was almost undoped silicon with hole concentration of about 10^15^ cm^−3^. Source and drain contacts were highly doped to form good ohmic contacts. For the connection to electronics, aluminum contacts were patterned using a lift-off process. Finally, chips were passivated with polyimide layer (PI) to protect metal feedlines from liquid environment. Figure 1 shows schematic pictures of the samples under study in the pH—sensor operating mode (a) and photo-receiver operating mode (b), and SEM picture of investigated NW is presented in Fig. 2.

## Results and Discussion

### Current-Voltage Characteristics and pH Sensitivity

**Fig. 3:**
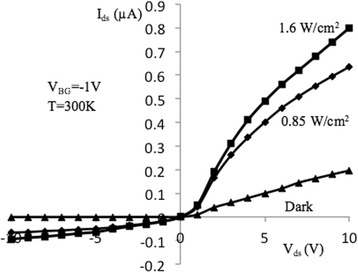
I–V characteristics of NW FET, measured at optical excitation (*V*_BG_ = − 1 V). Output current–voltage characteristics of NW FET sample with length *l* = 10 μm, measured in the dark and at excitation by the light specific power 0.85 and 1.6 W/cm^2^, at *T* = 300 K and *V*_BG_ = − 1 V

**Fig. 4:**
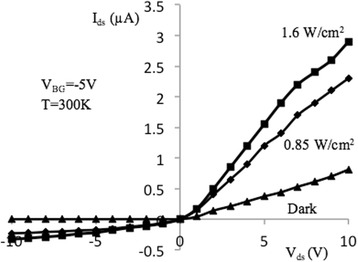
I–V characteristics of NW FET, measured at optical excitation (*V*_BG_ = − 5 V). Output current–voltage characteristics of NW FET sample with length *l* = 10 μm, measured in the dark and with excitation by the light specific power 0.85 and 1.6 W/cm^2^ at *T* = 300 K and *V*_BG_ = − 5 V

Figures 3 and 4 show source-drain current–voltage (I–V) characteristics of samples under study measured at back-gate voltages of − 1 and − 5 V, correspondingly. Characteristics were measured in the dark conditions as well as under specific power illuminations of 0.85 and 1.6 W/cm^2^ at room temperature. Light excitation is performed using incandescent lamps located at a distance of 15 cm from the sensor. The I–V dependencies demonstrate typical behavior which is similar to the metal-oxide-semiconductor FETs (MOSFETs) [24] since the samples under investigation have relatively large dimensions of *l* × *w* × *t* = (2 ÷ 10) × 10 × 0.05 μm (*l*, *w*, and *t* are the channel length, width, and thickness, correspondingly). I–V curves in Figs. 3 and 4 can be described as:

1 $$ {I}_{\mathrm{ds}}={I}_{\mathrm{ds},\mathrm{d}}+{I}_{\mathrm{ds},\mathrm{ph}}, $$

where *I*_ds, d_ and *I*_ds, ph_ are the dark and photo source-drain current components. Dark current can be described by the well-known expression for MOSFETs for *V*_ds_ ≤ *V*_gs_ − *V*_th_ [24]:

2 $$ {I}_{\mathrm{ds},\mathrm{d}}=\frac{w{\mu}_n{C}_{\mathrm{ox}}}{l}\left({V}_{\mathrm{gs}}-{V}_{\mathrm{th}}-\frac{V_{\mathrm{ds}}}{2}\right){V}_{\mathrm{ds}}. $$

Here, *C*_ox_ = *ε*_ox_/*t*_ox_ is the oxide layer capacitance per unit area, *ε*_ox_ and *t*_ox_ are the permittivity and thickness of the gate oxide layer, *μ*_*n*_ is the electron mobility, and *V*_ds_, *V*_gs_, and *V*_th_ are source-drain, gate-source, and threshold voltages, correspondingly. The generation rate of photo-carriers is equal to *ηαN*_ph_, where *N*_ph_ = *W*/*hν* is the intensity of irradiation. At low injection levels and constant lifetime of holes, the concentration of photo-carriers will be $\Delta  p=\eta \alpha {\tau}_p\frac{W}{h\nu}$ [25]. The carriers drift at applied voltage *V*_ds_. In this case, the photocurrent can be represented as:

3 $$ {I}_{\mathrm{ds},\mathrm{ph}}={A}_{\mathrm{ch}}e{\mu}_p\Delta  p\frac{V_{ds}}{l}={A}_{\mathrm{ch}}e{\mu}_p\eta \alpha {\tau}_p\frac{W}{h\nu}\frac{V_{\mathrm{ds}}}{l}. $$

Here, *A*_ch_ = *wt* is the current channel cross-section area, *e* is the electron charge, *∆p* and *μ*_*p*_ are the concentration and mobility of excess photo-carriers (holes), *α* the illumination absorption coefficient, *η* the quantum yield, *τ*_*p*_ the hole’s lifetime, *hν* the photon energy, and *W* the illumination specific power in [W/cm^2^].

In Eq. (3), we assumed that the electric field strength is uniformly distributed along the channel length and the value of *A*_ch_ slightly varies along the length of the channel due to high channel conductivity. It should be noted that this assumption is valid in the main part of the channel, which is far from source and drain contacts.

**Fig. 5:**
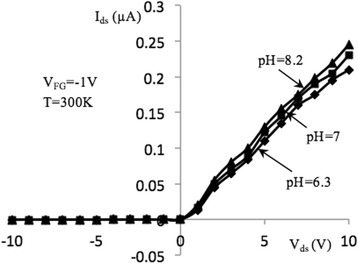
I–V characteristics of NW FET, measured in different pH solutions (*V*_FG_ = − 1 V). Output current–voltage characteristics of NW FET with length, *l* = 10 μm, measured in the dark and pH concentrations: 6.3, 7, 8.2 at *T* = 300 K, V_BG_ = − 5 V, and *V*_FG_ = − 1 V

**Fig. 6:**
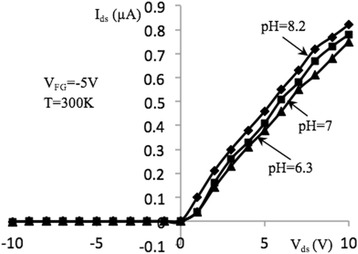
I–V characteristics of NW FET, measured in pH solutions (*V*_FG_ = − 5 V). Output current–voltage characteristics of NW FET with length, *l* = 10 μm, measured in the dark and pH concentrations 6.3, 7, 8.2 at *T* = 300 K, *V*_BG_ = − 5 V, and *V*_FG_ = − 5 V

At low voltages *V*_ds_, the source-drain current *I*_ds_ grows approximately linearly with voltage. With increasing light specific power, the magnitude of the *I*_ds_ increases. Figures 5 and 6 show I–V curves of the investigated device at the several front gate voltages (*V*_FG_ =  − 1 V, − 5 V) measured in an aqueous solution with pH = 6.2, 7, and 8.3. We can see that increasing the pH value results in the increase of the channel current,*I*_ds_*.* This is in a good agreement with model of the solution contact with the oxide layer surface, then on the oxide/solution interface caused hydroxyl groups SiOH. Concentration and behavior of those hydroxyl groups depend on value of the pH. The case when the surface is not charged is called zero charge point. For the SiO_2_ dielectric layer, the point is reached at pH = 2.2. At the pH values lower than 2.2, the oxide surface is charged positively; at higher values of the pH, oxide surface is charged negatively. In the case of buffer solution with pH = 7, silicon oxide surface charge will be charged negatively. Therefore, at the applied negative gate potential, the absolute value of the negative charge on the surface oxide increases. As a result, the majority carrier concentration increases in the current channel (holes in p-Si) and thus channel current increases.

Figures 5 and 6 show the I–V characteristics of the Si NW structures working in biochemical sensing mode. Measurements were performed four times for each pH value. The repeatability was within 7%. In [26], pH sensitivity of the biochemical sensors was introduced as

4 $$ {R}_{\mathrm{pH}}=\frac{R_{\mathrm{ch}}\Delta  {I}_{\mathrm{ds}}}{\Delta  \mathrm{pH}}. $$

Here, *∆I*_ds_ and ∆pH are the elementary changes in *I*_ds_ and pH. Note that pH sensitivity is the measurable value. In the solution medium with the increased pH value, the source-drain current increases. This allows the registration of the pH variation in any bio liquids (within the solution range relevant to physiological solutions) with high accuracy. For example, for *V*_BG_ =  − 5 V at the *V*_ds_ = 5 V, the sensitivity is equal to *R*_pH_ ≈ 56.4 mV/pH. At the *V*_BG_ =  − 5 V, the pH sensitivity grows up to 59.3 mV/pH and approaches the Nernst limit 59.5 mV/pH [24]. The pH sensitivity grows with increase of back-gate voltage. For example from Figs. 5 and 6 at *V*_ds_ = 8 V, we obtained the ratio ${\left({R}_{\mathrm{pH}}\right)}_{V_{\mathrm{BG}}=-5\ \mathrm{V}}/{(R)}_{V_{BG}=-1\ \mathrm{V}}\approx 5.17$, i.e., approximately five times improved sensitivity.

### Low-Frequency Noise Spectra and Features Caused by Irradiation and pH Changes

**Fig. 7:**
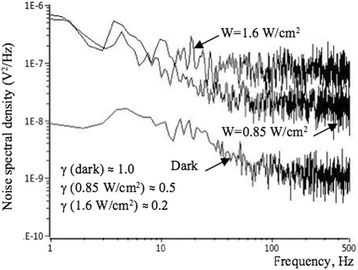
Noise spectra of NW FET, measured at optical excitation. Spectral dependence of LF noise, measured for NW FET sample with *l* = 10 μm under illuminations: 0.85 W/cm^2^, 1.6 W/cm^2^, and in the dark; *V*_BG_ = − 1 V, *T* = 300 K

The noise spectra of Si NW structures were measured at the constant current in the ohmic mode. Figure 7 shows the drain current noise power spectral density measured in dark conditions as well as under irradiation with applied back-gate voltage of *V*_BG_ =  − 1 V at *I*_ds_ = 0.1 μA. Noise spectra, measured in dark, demonstrate 1/*f*^*γ*^ noise behavior with noise parameter equal to *γ* = 1. Low-frequency (LF) noise level rises with the increase of the light irradiation intensity. The increase of the illumination intensity results in the growth of the major carrier’s concentration. This in turn causes the growth of mobility fluctuations in the channel because of increased interaction and scattering rates as result of scattering, first, between carriers, and second, between the carriers and acoustic phonons, as well as on different impurity traps [27].

Since the noise measurements were performed at the constant current in the ohmic mode, the channel resistance linearly changes with the applied voltage *V*_ds_. As it is known, the 1/f-noise spectral density *S*_*V*_ is proportional to the voltage in power 2:

6 $$ {S}_V=\frac{\alpha_{\mathrm{H}}{V}_{\mathrm{ds}}^2}{N{R}_{\mathrm{ch}}^2{f}^{\gamma }}=\frac{\alpha_{\mathrm{H}}{V}_{\mathrm{ds}}^2}{p\Omega {R}_{\mathrm{ch}}^2{f}^{\gamma }}=\frac{\alpha_{\mathrm{H}}{V}_{\mathrm{ds}}^2}{R_{\mathrm{ch}}^2{f}^{\gamma }}\frac{e{\mu}_p\rho }{A_{\mathrm{ch}}l}=\frac{\alpha_{\mathrm{H}}{V}_{\mathrm{ds}}^2}{f^{\gamma }}\frac{e{\mu}_p}{l^2}\frac{1}{R_{\mathrm{ch}}}\propto \frac{1}{R_{\mathrm{ch}}},\kern1.75em \frac{f^{\gamma }{S}_V}{V_{\mathrm{ds}}^2}\propto \frac{1}{R_{\mathrm{ch}}}. $$

Here, *α*_H_ is the Hooge parameter, *R*_ch_ is the current channel resistance; Ω = *A*_ch_*l* is the volume of the current channel; *ρ* is the channel specific resistance. The decrease of the channel resistance leads to growth of the noise spectral density. At the light excitation of nanowire FET sample with power *W*, we have:

7 $$ {\displaystyle \begin{array}{l}{S}_{V,L}=\frac{\alpha_{\mathrm{H}}{V}_{\mathrm{d}\mathrm{s}}^2}{NR_{\mathrm{ch}}^2{f}^{\gamma }}=\frac{\alpha_{\mathrm{H}}{V}_{\mathrm{d}\mathrm{s}}^2}{p\Omega {R}_{\mathrm{ch}}^2{f}^{\gamma }}=\frac{\alpha_{\mathrm{H}}{V}_{\mathrm{d}\mathrm{s}}^2}{\Omega {f}^{\gamma }}\frac{1}{p{\left(\rho l/{A}_{\mathrm{ch}}\right)}^2}=\frac{\alpha_{\mathrm{H}}{V}_{\mathrm{d}\mathrm{s}}^2}{\Omega {f}^{\gamma }}\frac{A_{\mathrm{ch}}^2{\sigma}^2}{pl^2}=\frac{\alpha_{\mathrm{H}}{V}_{\mathrm{d}\mathrm{s}}^2}{A_{\mathrm{ch}}{lf}^{\gamma }}\frac{A_{\mathrm{ch}}^2{e}^2p{\mu}_p^2}{l^2}=\\ {}\kern11.5em =\frac{\alpha_{\mathrm{H}}{V}_{\mathrm{d}\mathrm{s}}^2}{f^{\gamma }}\frac{A_{\mathrm{ch}}}{l^3}{e}^2{\mu}_p^2\left({p}_{\mathrm{d}}+\Delta p\right)=\frac{\alpha_{\mathrm{H}}{V}_{\mathrm{d}\mathrm{s}}^2}{f^{\gamma }}\frac{A_{\mathrm{ch}}}{l^3}{e}^2{\mu}_p^2\left({p}_{\mathrm{d}}+{\eta \alpha \tau}_p\frac{W}{h\nu}\right)\end{array}} $$

Here, *p*_d_ is the concentration of holes in the dark conditions and *σ* is the specific conductivity. The noise level increases proportionally to the intensity of the illumination.

We calculate values of the noise parameter *γ*, using the curves presented in Fig. 7. The following parameters are obtained for samples, measured in dark and at light excitation of different powers:

*γ*(dark) ≈ 1.0, *γ*(0.85 W/cm^2^) ≈ 0.5, and *γ*(1.6 W/cm^2^) ≈ 0.2.

Under irradiation, the value of the noise parameter *γ* decreases. This can be explained as follows. With increasing light power, the conductivity of the current channel increases. As a result, the effective lifetime of minority carriers *τ*_ef_ rises and reaches values *τ*_ef_ ≥ (10^−3^ ÷ 10^−2^) s. Once the electron-hole pairs are generated by absorption in silicon, several recombination mechanisms have to be considered. These processes occur in parallel, and the rate of recombination is the sum of rates corresponding to the individual process. Various lifetimes are associated with different recombination mechanisms. Тhe carrier effective lifetime has to be determined by the carrier’s surface, radiative, volume (bulk), and the Auger recombination lifetimes. It is known that the radiative lifetime is inversely proportional to the carrier density, and the Auger lifetime is inversely proportional to the carrier density squared [28]. The bulk recombination lifetime is determined by the Shockley-Read-Hall recombination mechanism. It is constant for low-level carrier densities, and it increases for high injection level [29–31]. It is known that surface recombination lifetime is proportional to the rate of surface recombination and inversely proportional to the thickness of the sample [29, 32, 33].

The behavior of the effective lifetime will be complex, depending on the nonequilibrium carrier’s density and mechanisms of recombination. As the carrier density increases, the effective lifetime can be either constant or decreasing function [29]. In the nanowires at high ratio of surface to volume, the surface interface states play more important role and their contribution dominates. Furthermore, it can surpass other types of recombination. On the other hand, at the moderate level of carrier’s density, bulk recombination lifetime can also increase. For our case of silicon NW carrier's, effective lifetime is determined basically by the surface and bulk recombination and increase with carrier's density growth.

As it is known, generation-recombination (g-r) noise has the Lorentzian shape [19, 34]:

8 $$ {S}_{V,g-r}\sim \frac{1}{1+{\left(2\pi f{\tau}_{\mathrm{ef}}\right)}^2}. $$

Here, *f* is the frequency. It is clear that the section of the plateau on dependence *S*_*V*, *g* − *r*_(*f*) is determined by the condition

9 $$ 2\pi {f}_c{\tau}_{\mathrm{ef}}\le 1, $$

where *f*_*c*_ is the characteristic frequency. It should be noted that with the increase of electrons lifetime the value of the cutoff frequency *f*_*c*_ decreases. The characteristic frequency of the g-r noise shifts to the low-frequency region. Since the conductivity *σ* and lifetime *τ*_*n*_ increase with increasing illumination power, the *f*_*c*_ decreases with increasing *W*, correspondingly:

10 $$ {f}_c\propto \frac{1}{\tau_{\mathrm{ef}}}\propto \frac{1}{W}. $$

The g-r processes lead to the screening of the 1/f-noise component under the g-r noise plateau. The fact explains the decrease in the value of the noise parameter *γ* with increasing illumination power.

**Fig. 8:**
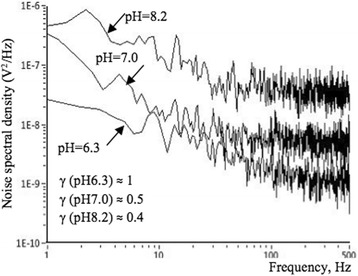
Noise spectra of NW FET, measured in different pH solutions. Spectral dependence of LF noise for NW with length, *l* = 10 μm, measured at *T* = 300 K and several pH values: 6.3, 7.0, and 8.2 at *V*_BG_ = − 5 V, *V*_FG_ = − 1 V

Figure 8 illustrates spectral dependence of the LF noise power spectrum of Si NW FET sample, measured at the *V*_*FG*_ =  − 1 V, *I*_ds_ = 0.1 μA in solution at the several pH values: 6.3, 7.0, and 8.2. Noise parameter decreases with the increasing of the pH value: *γ*(pH = 6.3) ≈ 1.0; *γ*(pH = 7.0) ≈ 0.5; *γ*(pH = 8.2) ≈ 0.4. Тhe slopes were calculated in the range from 10 to 500 Hz. LF noise level increases and its slope decreases with increase of the pH value. The increase in pH value leads to a decrease in channel resistance, which is caused by the accumulation of negative charges at the semiconductor-oxide interface. Decreasing of the slope of *S*_*V*_(*f*) dependence with pH increasing can be explained taking into account the effect of the channel conductivity increasing.

### Effects of the Channel Length

**Fig. 9:**
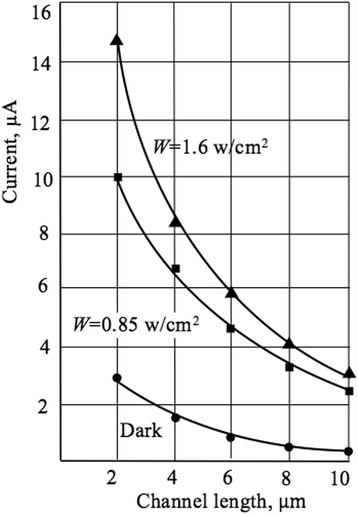
Channel current of NW FET vs length. Plot of channel current as a function of channel length. *V*_BG_ = − 5 V, *V*_ds_ = − 5 V, *R*_ch_ = 1.26 MΩ

**Fig. 10:**
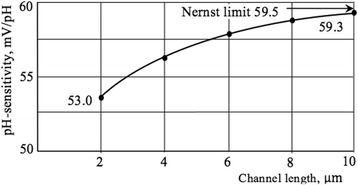
pH sensitivity vs channel length. Plot of pH sensitivity as a function of channel length. *V*_FG_ = − 10 V, *V*_BG_ = − 5 V, *V*_ds_ = − 5 V, *R*_ch_ = 1.26 MΩ

In this section, we present the results of the influence of the current channel length on the transport mechanisms, pH sensitivity, and also on the behavior of LF noise of the Si NW-based sensors. The magnitude of the current is inversely proportional to the length of the current channel, which justifies the application of the drift approximation for transport mechanism, as well as the assumption of a uniform distribution of the electric field strength along the length of the current channel (Fig. 9). The influence of light excitation leads to an increase in the magnitude of the source-drain current. The pH sensitivity increases with the current channel length and tends to the Nernst limit of 59.5 mV/pH (Fig. 10), which is in good agreement with values obtained for micro-size sensors [27]. Our results support also observations of the pH sensitivity behavior obtained for NW samples with different geometries [6]. The length effect studied systematically in our work can be explained as follows. Since the length of the channel *l* decreases, the area of the pH-sensitive surface decreases, and consequently the number of measurable H^+^ ions in the aqueous solution decreases. According to Eq. (2), the current *I*_ds_ increases with decreasing *l*, which leads to a decrease in the resistance of the current channel at constant voltage *V*_ds_. As the resistance of the channel *R*_ch_ decreases, its modulation is hampered under the influence of the H^+^ ions; hence, the pH sensitivity decreases.

**Fig. 11:**
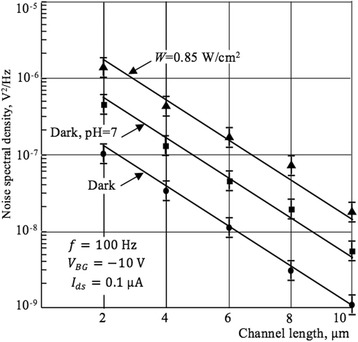
Noise spectral density vs current channel length. Plot of noise spectral density as a function of current channel length. For pH measurements *V*_FG_ = − 10 V

Figure 11 illustrates the LF noise spectral density dependence on the length of the current channel.

These curves are plotted using the spectral dependences of the LF noise measured for Si NWs with different lengths in the dark conditions, under illumination with an intensity of 0.85 V/cm^2^, and in an aqueous solution with a pH = 7. Calculated value of the slope of the parallel curves (Fig. 10) is equal to log(500/10) ≈ 2.7. This value is close to the value (equal to 3) obtained theoretically using Eqs. (6) and (7), *S*_*V*_ ∝ *l*^−3^ with error about 10%. The results demonstrate that theoretically predicted S_V_(*l*) dependencies are in good agreement with measured characteristics taking into account relatively high level of thermal noise. According to Eq. (6), scaling the channel length *l* down leads to decrease of resistance and current increase, which corresponds to increase of charge carriers in the channel. This, in turn, results in increased interaction of charge carriers with traps on the interface between silicon and dielectric layer. Thus, the noise level increases, which is also confirmed by experimental dependences (see Fig. 11).

The non-Nernstian pH-response of SiO_2_-gated FET-based sensors has been a major topic since the introduction of the ion-sensitive FET (ISFET) concept. The sensitivity of the SiNR FET sensor to changes in pH can be quantified by measuring the shift of the threshold voltage of the device and is defined by the Nernst equation [35]:

$$ \frac{\delta {\Psi}_0}{\delta \mathrm{pH}}=-2.3\frac{kT}{q}\alpha \le 59\ \frac{mV}{\mathrm{pH}} $$,

where *δ*Ψ_0_ is the potential at the surface. The dimensionless parameter a which depends on the intrinsic buffer capacity of the oxide surface and the differential double-layer capacitance can be a value between 0 and 1.

Changes in the pH of the solution induce variations in the surface charge density and surface potential. It leads to a change in the NR channel conductance. In general, sensitivity is defined as the largest possible output response to a certain biological event. The pH sensitivity of BioFETs arises from the acid/base reactions at the oxide/electrolyte interface and the maximum pH response achievable by a conventional ISFET is the Nernst limit of 59 mV/pH. Over the years, there have been numerous reports [36–45] on devices with near Nernstian. The high sensitivity was achieved either by optimization of the intrinsic device transfer characteristics (such as lowering of the subthreshold swing or by tuning the gate potential) or by chemical surface modifications. Decreasing silicon thickness leads to higher surface charge sensitivity [45]. In [6], it is shown that at an optimum thickness of 30 nm the sensitivity reaches maximum value, and for a thicker device layer the pH response decreases and the largest response is obtained from the widest NR FET with the highest surface area. The most popular platform for chemical modification of SiO_2_ surface is chemisorption of a few nanometer thick self-assembled monolayers [46], not only to enhance the pH sensitivity of Si/SiO_2_ gated nanosensors [47], but also because biomolecules such as proteins [48] or DNA [49], which can be coupled to the other functional end of certain monolayers. Authors of Ref. [50] discussed the results concerning the functionalization and modification of SiNW FET sensors.

## Conclusions

Silicon nanowire FET biochemical sensors of various lengths were fabricated. The static dark and light-illuminated I–V curves as well as the behavior of these sensors in an aqueous solution with different values of pH are investigated. The static dark I–V dependencies demonstrate FET behavior. With increasing light intensity, the source-drain current grows because of the increase in the conduction of the current channel. The pH sensitivity increases with the increasing of the back-gate voltage and approaches to 59.5 mV/pH. The magnitude of the channel current is approximately inversely proportional to the length of the current channel and the pH sensitivity increases with increase of channel length approaching to the Nernst limit value, indicating that larger area devices are more suitable for the pH sensing.

The spectral density of the LF noise increases both under the action of the pH solution and the illumination, and in both cases, the frequency dependence of the noise is weakened and the value of the noise parameter γ decreases. With increasing of the pH value and illumination power, the 1/f-noise is screened by the g-r plateau. The characteristic frequency of the g-r noise component decreases with increasing illumination power. LF noise level increases and its slope decreases with increase of the pH value. It is shown that the measured value of the slope of noise spectral density dependence on the current channel length is 2.7 that is close to the theoretically predictable value 3 within 10% error.

## Abbreviations

FETs: Field-effect transistors
LF: Low-frequency
NWs: Nanowires
TMAH: Tetramethylammonium hydroxide
